# Review of Atlas of Human Anatomy, 4^th ^Edition, by Frank Netter

**DOI:** 10.1186/1477-7800-4-28

**Published:** 2007-12-16

**Authors:** Gurpreet Singh Ranger, Chung Lim

**Affiliations:** 1 Department of General Surgery, Queens Hospital, Romford, Essex, UK; 2 Department of Vascular Surgery, Charing Cross Hospital, London, UK

## 

Frank Netter's rendering of the different parts of the human body are familiar to medical students, physicians and surgeons the world over – moreover, many patients have seen them in consulting rooms. Netter produced over 4000 high quality illustrations over his career, and this atlas represents anatomical paintings from the Netter collection, now extensively revised and updated by an experienced team of consulting editors – they deserve a mention for their input into this book – Jennifer K. Brueckner, Stephen W. Carmichael, Thomas R. Gest, Noelle A. Granger, John T. Hansen and Anil H. Walji

This book in hardback is 23 cm × 29 cm, and contains a CD ROM with plates from the Atlas (Figure 1). There are no page numbers in this book – only plates (548 in total), which gives it a feel of a work of art rather than a standard anatomy textbook.

**Figure 1:**
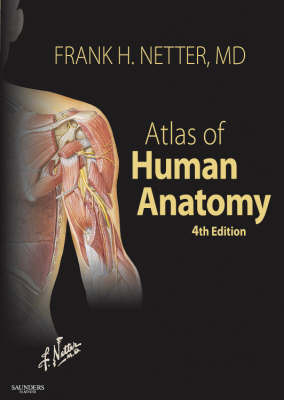
Front Cover of the Staging Atlas.

It is logically divided into sections corresponding to the different regions of the body, and nearly every single page of this book contains a detailed and beautiful anatomical illustration (Figure 2). These have been brought into the 21^st ^century with a multitude of radiological images, for example MRI and CT scans, and access to supplemental material on the worldwide web. This is a considerable achievement by the editorial team.

**Figure 2:**
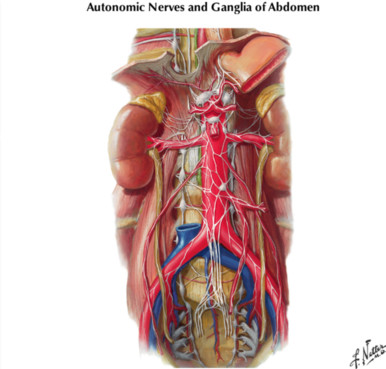
Plate from the Atlas – Autonomic Ganglia In The Abdomen.

I found this book invaluable in illuminating my understanding of complex areas of human anatomy – for example the pelvic floor and nervous systems.

Medical students and surgical trainees without doubt would benefit the most from this book, but it is a must for all health care professionals to have on their bookshelf.

## Competing interests

The author declares that he has no competing interests.

